# A Preliminary Study on the Accuracy of MRI-Guided Thalamic Infusion of AAV2-GFP and Biodistribution Analysis Using Cryo-Fluorescence Tomography in Nonhuman Primates

**DOI:** 10.3390/pharmaceutics17091167

**Published:** 2025-09-06

**Authors:** Ernesto A. Salegio, Reinier Espinosa, Geary R. Smith, David Shoshan, Matthew Silva, Eli White, Jacob McDonald

**Affiliations:** 1 ClearPoint Neuro Inc., Solana Beach, CA 92075, USA; ernestosalegio@gmail.com (E.A.S.); csmith@clearpointneuro.com (G.R.S.); dshoshan@clearpointneuro.com (D.S.); 2 Envol Biomedical, Immokalee, FL 34142, USA; reinier.e@envolbio.com; 3 EMIT Imaging, Inc., Natick, MA 01760, USA; matt.silva@emitimaging.com (M.S.); eli.white@emitimaging.com (E.W.)

**Keywords:** intraparenchymal infusion, thalamus, AAV2, MRI-guided delivery, cryo-fluorescence tomography, biodistribution, brain, primates

## Abstract

**Background:** Adeno-associated viral (AAV) vectors are the leading platform for gene therapy, but common delivery routes show limited spread to distal cortical structures, hence the utility of direct, intrathalamic infusions for broader transgene distribution. In this preliminary study, we recapitulate previous studies targeting the thalamus as a conduit to achieve cortical transgene spread and showcase novel data evaluating biodistribution of a green fluorescent protein (GFP) using cryo-fluorescence tomography (CFT). For the first time in nonhuman primates (NHPs) and coupled with magnetic resonance imaging (MRI)-guidance, we demonstrated the application of CFT as a powerful tool to map out vector distribution in the NHP brain. **Methods:** Briefly, a single thalamic infusion was performed in African green monkeys using ClearPoint’s navigational platform to deliver an AAV serotype 2 vector containing a GFP payload. Transgene biodistribution was assessed in the left and right hemispheres using CFT and histological analysis, respectively. **Results:** Infusions were successfully performed with sub-millimetric target accuracy and with minimal error, achieving ~86% thalamic coverage with the largest infusion volume. Histology confirmed the presence of the GFP transgene, with the strongest signal in the cerebral gray/white matter and internal capsule, while CFT allowed for the three-dimensional detection of the transgene starting at the site of infusion and spreading to multiple cortical regions. **Conclusions:** These findings suggest that by combining MRI-guided technology with CFT imaging, it is feasible to map whole-brain gene biodistribution in NHPs. This proof-of-concept study bridges the gap between cellular microscopy and MRI-guidance to provide a complete picture of disease and treatment with clinical applicability.

## 1. Introduction

The adeno-associated viral (AAV) vector is currently the most widely used and well-established platform for gene therapy [1]. Recent advances in AAV-mediated gene therapy have led to significant therapeutic breakthroughs in rare genetic disorders, as evidenced by multiple recent FDA/EMA approvals [2]. While less invasive routes such as cerebrospinal fluid (CSF) and intravenous delivery are commonly employed, preclinical studies in nonhuman primates (NHPs) suggest limited transduction efficiency in deeper brain structures, such as the thalamus or striatum, and reduced therapeutic efficacy in certain neurodegenerative diseases [3]. Additionally, delivery of therapeutics into the CSF has been reported to lead to global, nonspecific gene exposure throughout the central nervous system (CNS) including brain, spinal cord, and dorsal root ganglia [4]. Conversely, a direct intraparenchymal infusion into the brain can keep the exposure of the therapeutic within the parenchyma, bypass the blood–brain barrier, and concomitantly minimize neurotoxicity and systemic exposure [5,6]. It also provides neurosurgeons with a more predictable pattern of therapeutic distribution and a greater level of control during the overall procedure. Control can be crucial when targeting anatomical regions of known axonal projections, such as the putamen and thalamus, and/or when trying to maximize coverage of a preselected target.

In fact, intrathalamic infusions are particularly promising due to the extensive connectivity of the thalamus with the cerebral cortex, thus enabling transgene transport to multiple brain regions [7,8,9]. Safety in performing such infusions has been demonstrated in multiple nonclinical models, including NHPs, with no untoward side effects noted [8,9,10]. However, as with any direct brain infusion, it is understood that there may be some risks associated with this type of procedure and that is why it is important to utilize reliable delivery methods that offer consistent sub-millimetric accuracy and little-to-no error during targeting. Achieving such accuracy warrants the use of precise, image-guided techniques to minimize any type of complications, neurological or otherwise. Challenges related to therapeutic delivery are not only associated with the surgical procedure itself, but also with the ultimate biodistribution of the therapeutic, which clinically has been shown to result in poor therapeutic efficacy due to inaccurate tip placement within the anatomical target, insufficient coverage, and/or low infusion volume [11,12,13]. Therefore, to ensure a strong correlation between optimum therapeutic delivery and desired target coverage, it is necessary to perform the delivery under real-time magnetic resonance imaging (MRI) and then attempt to map the expected biodistribution of the therapeutic.

The main limitation of MRI-guidance is the inability to monitor for therapeutic expression post-infusion. On the other hand, cryo-fluorescence tomography (CFT) is an emerging 3D imaging technique that enables high-resolution visualization of drug distribution and transgene expression in whole animals or intact organs, such as the brain [14]. Pioneered by Wilson and colleagues [15], CFT involves embedding the entire specimen in optimal cutting temperature (OCT) compound, then robotically sectioning it into slices. After each cut, white-light and multi-spectral fluorescence images are captured and reconstructed into detailed 3D volumes. By bridging the resolution gap between in vivo imaging and histology, CFT allows unbiased mapping of transgene biodistribution while minimizing the need for labor-intensive histological methods [14]. To date, CFT imaging of the brain in gene therapy applications has only been demonstrated in rodents [16].

This small proof-of-concept study aimed to assess the feasibility of combining the precision of MRI-guided technology and CFT to determine the ultimate spread of a green fluorescent protein (GFP) AAV serotype 2 (AAV2) vector following infusion into the thalamus of NHPs. Histological analysis of vector distribution confirmed, for the first time, the successful application of CFT technology in an NHP brain following the targeted infusion of a viral vector. This approach enabled detailed characterization of vector spread and allowed for assessment of concordance between imaging and more traditional histological data. These findings provide a novel framework for validating targeted gene delivery techniques and improving our understanding of viral vector distribution in the primate CNS.

## 2. Materials and Methods

### 2.1. Ethics Statement

The research conducted in this study complied with the USDA Animal Welfare Act (9 CFR Parts 1–3) and adhered to the Guide for the Care and Use of Laboratory Animals. This study was conducted in Envol Biomedical’s animal facilities, which are fully accredited by the Association for Assessment and Accreditation of Laboratory Animal Care (AAALAC) International. All procedures were conducted under a protocol approved by the Institutional Animal Care and Use Committee (IACUC protocol: ENV2403).

### 2.2. Animals

Two healthy, male, non-naïve, African green monkeys (*Chlorocebus aethiops*; 4–6 kg body weight; 3–5 years) were used and housed at Envol Biomedical. Before and/or after dosing, animals were individually housed in stainless steel cages in a room maintained on a 12 h light/12 h dark cycle under controlled temperature. Animals were fasted prior to dosing, otherwise food and potable water were available *ad libitum*. Given the small number of animals in this study, no randomization or blinding or exclusion criteria were implemented.

### 2.3. Test Article

The test article, AAV2-GFP (lot: RVC0336; dose level: 1.66 × 10^13^ vg/mL) encoding enhanced GFP under the control of a cytomegalovirus (CMV) promoter, was packaged at the Research Vector Core at the Children’s Hospital of Philadelphia and was stored at minus 60 °C. On the day of dosing, frozen formulations were thawed at 4 °C, then mixed at a 1:250 ratio with Gadoteridol (Bracco Diagnostics Inc., Princeton, NJ, USA) by gentle inversion. Dosing vials were allowed to reach ambient temperature prior to administration.

### 2.4. MRI-Guided Intracerebral Infusion

The delivery system and procedure have previously been described [17,18,19,20]. Briefly, animals were sedated with intramuscular ketamine (10 mg/kg, Covertus, Immokalee, FL, USA) and dexmedetomidine (0.03 mg/kg Covertus, Immokalee, FL, USA), intubated, and placed on inhaled isoflurane (1–3%). Subjects were placed supine with head secured to ClearPoint’s MRI-compatible stereotactic frame (ClearPoint Neuro Inc., San Diego, CA, USA). A baseline high-resolution anatomical MRI scan was acquired for target identification and surgical planning. Upon selecting the appropriate trajectory to reach the pre-selected anatomical target, ClearPoint’s orchestra frame was attached to the MRI table and then ClearPoint’s SmartFrame^®^ array (ClearPoint Neuro Inc., San Diego, CA, USA) was mounted on the skull using titanium screws. A hand twist drill with a drill bit of 4.5 mm in diameter was passed through the guide stem of the array frame to create the desired percutaneous entry point. Upon confirmation of a clear path through the bone and into the brain, a pre-primed SmartFlow^®^ cannula (ClearPoint Neuro Inc., San Diego, CA, USA) containing the test article was inserted to target and the infusion was initiated. AAV2-GFP co-mixed with gadoteridol was infused under near real-time MRI guidance into the thalamus, starting with the right hemisphere and then the left hemisphere. The former hemisphere received a more focal infusion with a total volume of approximately 85 μL, whereas the contralateral hemisphere received approximately 170 μL (i.e., double the volume). Infusions were initiated at ~1 μL/min and ramped up to ~5 μL/min until the completion of the infusion. Immediately following dosing, a high-resolution scan was acquired, and the wound site was closed. Animals were monitored throughout the procedure via electrocardiogram (ECG; Invivo Expression Cat. #989803162711, Invivo Corporation, Orlando, FL, USA). Given that this was as a small proof-of-concept study, no control group was included and both animals underwent the same surgical procedure.

### 2.5. Clinical Observations

Pre- and post-operative medications for pain and distress included atipamezole (0.03 mL/kg, IM, Covertus, Immokalee, FL, USA), buprenorphine (0.01–0.03 mg/kg, IM, Covertus, Immokalee, FL, USA), Buprenorphine long-lasting (0.24 mg/kg, SC, Covertus, Immokalee, FL, USA), Carprofen (4.4 mg/kg, IM, Covertus, Immokalee, FL, USA), and Cefazolin (100 mg, IV, Patterson, Immokalee, FL, USA).

Cage-side observations were conducted on Day 1 (pre-dose) and once weekly thereafter until necropsy. Assessments included clinical signs such as gross motor, behavioral activity and visible changes in appearance. Body weight and food consumption were also recorded weekly throughout the study.

### 2.6. Tissue Processing

At the time of necropsy (28 ± 2 days post infusion), a transcardial perfusion of cold saline was performed with no fixative. The right and left hemispheres were carefully separated by making a sagittal cut using blunt dissection. The right hemisphere was processed for histological analysis, while the left hemisphere was reserved for cryo-fluorescence tomography. Preparation for CFT involved freezing the hemisphere on dry ice until completely frozen and then shipping it to EMIT Imaging (EMIT Imaging, Natick, MA, USA) for CFT imaging. No other tissues were collected post perfusion.

### 2.7. Histological Staining and Analysis (Chromogenic Detection)

Post-fixed serial coronal slabs of a hemibrain were processed by Experimental Pathology Laboratories (EPL^®^ Inc., Sterling, VA, USA). At EPL, brain slabs were processed for paraffin embedding, sectioned, immunohistochemically stained against GFP (rabbit anti-GFP, Abcam Cat# ab183735, Waltham, MA, USA), counterstained with hematoxylin for 5 min and visualized with 3, 3′-diaminobenzidine (DAB; Leica Biosystems, Cat# AR9432, Deer Park, IL, USA). Anti-GFP labeling was mapped with corresponding regions in a stereotaxic macaque brain atlas [21]. The location and intensity of anti-GFP labeling was rated using a semi-quantitatively 4-point scale as follows: 1—rare to sparsely scattered axons/cells within the region (approx. <5%); 2—occasional axons/cells within the region (approx. 5–10%); 3—frequent axons/cells within the region (approx. 11–50%); 4—majority of axons/cells within the region (>50%).

### 2.8. Cryo-Fluorescence Tomography

The frozen brain was embedded sagittally in a block of Dark OCT 31™ measuring 12 cm × 9 cm × 5 cm designed for the Xerra™ CFT system (EMIT Imaging, Natick, MA, USA). Following autofocusing and trimming, the CFT imaging process of serial anatomical and fluorescence block-face imaging was initiated. CFT imaging was performed with a 12 mega-pixel camera, with an image size of 4096 × 3008 pixels, resulting in a 30 µm in-plane pixel size, and to maintain isotropic voxels, serial sectioning was performed at 30 µm thickness. The GFP signal was detected using excitation at 470 nm and filtered detection at 511 nm (20 nm bandpass). Due to the uncertainty regarding signal strength, five fluorescence images were acquired from 5 to 2500 milliseconds (ms).

During image acquisition, both fluorescent and anatomical images underwent corrections to ensure consistency and accuracy; a flat-field correction was applied to compensate for non-uniform illumination and detector response, a dark-field correction was applied to remove background noise and offset variations in the detector, and warping was performed to correct chromatic aberrations. These corrections align the fluorescence and anatomical images and ensure even illumination across the block, enhancing image quality for visualization and analysis.

The acquisition image resulted in image stacks (one anatomical and five fluorescence exposures per slice) suitable for visualization and analysis using Fiji/ImageJ [22] or similar software packages. The final image size after cropping unnecessary portions of the field of view was 2346 × 2274 × 1453 pixels, and the 50 ms exposure image was used for visualizations. Visual outputs included flythrough movies of both fluorescent and anatomical image stacks, along with representative axial slices and region-of-interest (ROI) overlays for both fluorescence channels.

### 2.9. MRI and 3D Volume Reconstruction

A 3T MRI scanner with a Siemens flex coil (3T Radiology & Research, Aventura, FL, USA) was used to acquire high-resolution, 3D longitudinal relaxation time-1-weighted (T1w) fast spoiled gradient echo scan to generate coordinates for cannula trajectory. MRI scans captured at the end of infusion were reconstructed using OsiriX MD software (v.14.1.2, Bernex, Switzerland). As previously reported, all ROIs, defined as areas of hyperintense signal detected on T1w images, were manually traced on each MRI slice using an automated ROI volume tool [19]. Since no direct comparisons were made between each of the infusions, no statistical analysis was required, and all data points were included (no exclusions).

## 3. Results

### 3.1. Performance of ClearPoint Navigational Software

**Figure 1:**
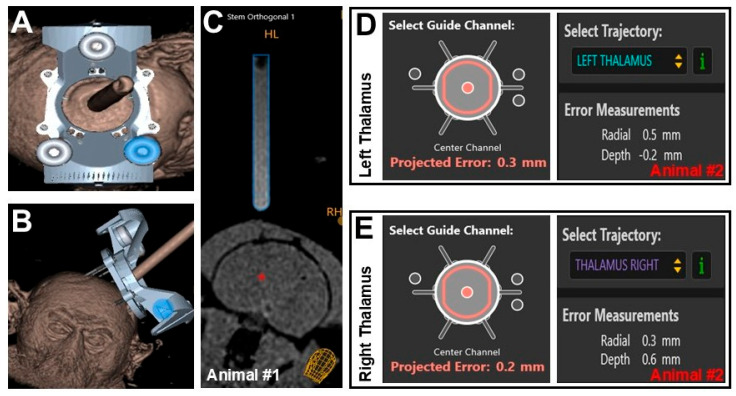
Alignment and targeted error using ClearPoint navigational software. Three-dimensional reconstruction of ClearPoint’s array frame mounted on the skull showing (**A**) top view and (**B**) frontal view of the device, as well as (**C**) an orthogonal view of the fluid filled stem in relation to the brain (Animal #1). Targeting channel showing projected error and actual error calculated by ClearPoint’s navigational software for (**D**) left and (**E**) right thalami (Animal #2). Red asterisk on Panel (**C**) corresponds to the approximate site of infusion.

MRI-guided intra-thalamic infusions were successfully performed without complications. The ClearPoint navigational software (version 3.0) automatically calculated targeting error, defined as the three-dimensional distance between the expected cannula tip location and the actual location marked by the operator on postinsertion imaging. Figure 1 illustrates the three-dimensional positioning of the array frame mounted on the skull including the 3-donut fiducial markers and a fluid-filled stem containing MRI contrast agent (Figure 1A–C). These components are used by the software to triangulate spatial positioning of the frame in relation to the patient. Following completion of alignment steps, the fluid-filled stem is replaced with a targeting stem with 6 potential options (i.e., holes) for cannula insertion into the desired anatomy. In this particular example, the center channel was selected instead of the other auxiliary entries and the software generated a projected error with radial and depth errors applicable to cannula placement in situ (Figure 1D,E). No MRI-visible hemorrhages were observed during cannula placement, and no adverse events were noted post-infusion.

As summarized in Table 1, targeting accuracy was high across all procedures, demonstrating close concordance between planned and actual infusion trajectories. In animal #1 (right side only), the actual radial error was 0.6 mm as compared to the projected radial error of 0.7 mm with a 0.1 mm differential between actual and projected. Final depth error was −0.1 mm, which meant that the tip of the cannula was shallow by 0.1 mm. For animal #2, projected vs. actual radial error for the right side was 0.3 mm and 0.2 mm with a 0.1 mm error differential and a 0.6 mm error in depth. For the left side, a 0.5 mm vs. 0.3 mm radial error was observed between projected and actual with a 0.2 mm differential. The depth error for the left side was −0.2 mm.

### 3.2. AAV2-GFP Vector Distribution

**Figure 2:**
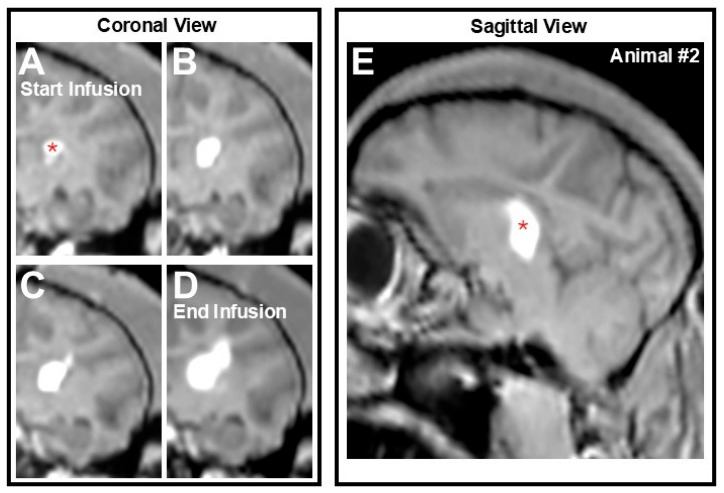
Infusate spread during real-time MRI guidance. Coronal view of infusate distribution within the left thalamus from (**A**) the start of the infusion, (**B**,**C**) during, and (**D**) until end of the infusion (i.e., acquisition of final MRI scan—Animal #2). (**E**) Sagittal view at the end of infusion showing approximately 86% coverage of the thalamus with an estimated infusion volume of 170 µL. Panels (**A**–**E**) are from the left hemisphere of Animal #2 and showcase a single intraparenchymal infusion. This animal also received an infusion into the contralateral hemisphere. Red asterisk on Panels (**A**,**E**) correspond to the approximate site of infusion.

All infusions were monitored in real time MRI-guidance and infusate distribution was detected by the presence of a hyperintense signal corresponding to the AAV comixed with the contrast agent. Animal #1 underwent a single intraparenchymal procedure in the right hemisphere, whereas animal #2 underwent a bilateral procedure whereby both hemispheres were targeted. Coronal images from Animal #2 captured throughout the infusion illustrate progressive and controlled spread of the AAV2-GFP vector within the left thalami from the start to the end of the infusion (Figure 2A–D). A post-infusion sagittal view of the region of interest demonstrates approximately 86% coverage of the left thalamus, with a total estimated infusion volume of 170 µL. Conversely, in the contralateral right hemisphere with a more focal volume of infusion of 85 µL, coverage of approximately 43% was achieved. In this particular figure, the largest infusion on the left hemisphere is shown, although as noted above this animal also received an infusion on the opposite hemisphere. All infusions were single, one-time infusions performed under convection-enhanced delivery (CED), evident by the radial infusion shape, a known hallmark of CED [20,23,24].

### 3.3. Biodistribution of GFP Transgene

**Figure 3:**
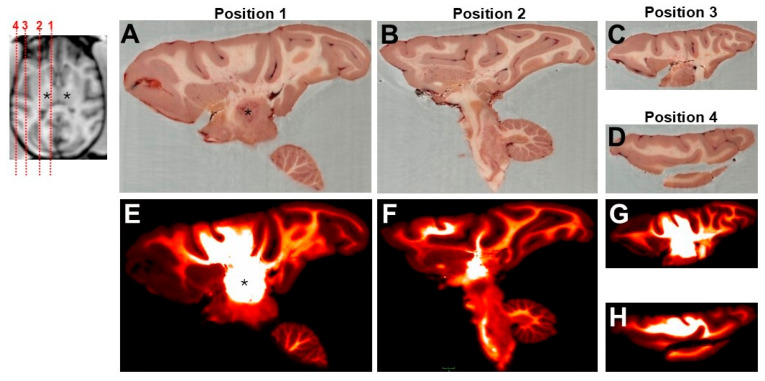
Biodistribution of AAV2-GFP using cryo-fluorescence tomography. Four representative positions along the sagittal plane of the left hemisphere were taken during brain slicing to demonstrate biodistribution of the GFP transgene under (**A**–**D**) brightfield and (**E**–**H**) fluorescence imaging. MRI insert shows estimated positioning of the four selected slices from the most medial position (**A**,**E**) to the most lateral position (**D**,**H**) in the brain, where Position 1 corresponds to Panels (**A**,**E**), Position 2 Panels (**B**,**F**), Position 3 Panels (**C**,**G**), Position 4 Panels (**D**,**H**). Note: location of cerebellum denotes posterior/occipital end of the brain. Black asterisk on the insert, Panels (**A**,**E**) correspond to the approximate site of infusion.

Post transcardial perfusion (28 ± 2 days post infusion), biodistribution of the transgene was assessed across four representative brain sections in the left hemisphere using CFT. As shown in Figure 3, the GFP signal was widely observed across the targeted region, with no abnormal pathology detected in any of the selected slices. Starting at position 1, which corresponds with panels A and E, showcases the spread of the transgene at the site of infusion (e.g., the thalamus) and can be seen extending dorsally throughout different parts of the overlying cortex, not only in gray matter but also in white matter. Transport is presumed to have occurred along thalamo-cortical projections, and this is most clearly seen at the level of the anatomy, corresponding to position 1 (i.e., the most medial brain slice shown here). The following panels B and F (position 2) demonstrate presence of the GFP transgene transported ventrally to the brainstem and more distally in the prefrontal cortex. The remaining panels C–H (positions 3, 4) reveal the full coverage of the transgene expression distributed to the most lateral part of the cortex (i.e., the most lateral brain slices shown here).

**Figure 4:**
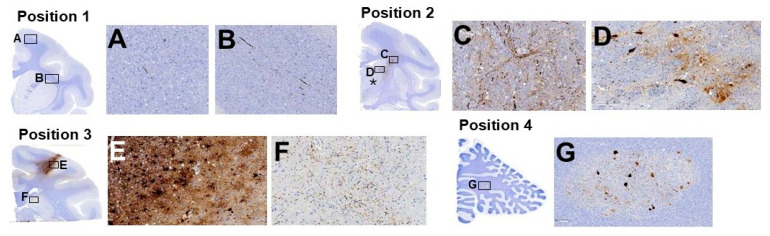
Detection of AAV2-GFP expression across the right hemisphere using histological staining after 85 µL infusion. Four representative positions along the coronal plane are shown (**A**–**G**), starting at the most rostral (**A**,**B**) to the most caudal position (**G**). Note: some leakage was observed during cannula withdrawal from the brain and was confirmed during histology, especially in position 3 (panel **E**). All panels (**A**–**G**) are from the right hemisphere of animal #2 and showcase a single intraparenchymal infusion. Black asterisk on Position 2 insert corresponds to the approximate site of infusion. Magnification: position 1 insert 0.4× and panels (**A**,**B**) 10×; position 2 insert 0.4× and panels (**C**,**D**) 20×; position 3 insert 0.5× and panel (**E**) 10× and panel (**F**) 20×; position 4 insert 0.6× and panel (**G**) 10×.

Biodistribution of the transgene was also assessed using histological analysis in the right hemisphere. This was the most focal infusion with approximately half of the total amount of volume delivered (85 µL) as compared to the contralateral hemisphere (170 µL), and it served as internal control to the AAV2-GFP expression. As shown in Figure 4, even with a smaller infusion volume, widespread transgene distribution was observed across all sampled regions, confirming the ability of this transgene to be transported distally following a single infusion. As importantly, the GFP transgene was found extending from the prefrontal cortex to the deep cerebellar nucleus. In fact, GFP transport was detected from approximately 4 mm rostrally and 25 mm caudally to/from the anterior commissure. Therefore, histologically, the GFP transgene distributed 36 mm along the rostral-caudal axis of the NHP brain after a single 85 µL infusion. At the end of the infusion during cannula withdrawal, some leakage of the infusate was noted along the trajectory of the cannula and this can be corroborated histologically (Figure 4E). However, this did not result in any adverse events for the animal.

As shown in Table 2, detection of the transgene was most prominently expressed in axons and neurons across multiple brain regions, with particularly strong GFP expression observed in the cerebral gray and white matter and internal capsule. Which is consistent with known tropism of the AAV2 vector backbone. Moderate expression was also noted in ependymal cells of the lateral ventricle.

## 4. Discussion

Effective biodistribution of therapeutics within the CNS is crucial when attempting to treat CNS-based disorders and one key consideration is the contribution of anatomical and functional circuits in the brain. Therefore, the main purpose of this study was to expand on previous studies and provide novel proof-of-concept data demonstrating the usefulness of multiple imaging modalities to monitor transgene expression in the brain. Earlier work targeting highly interconnected anatomical regions, such as the thalamus, revealed the benefit of targeting such anatomy to distribute therapeutics to a much larger volume of brain [7]. In our experience, to be able to perform such a procedure successfully, certain key factors must be considered, such as accuracy, the number of injections required to maximize coverage of the desired anatomy, and the ultimate distribution wanted. Interaction of these factors is governed by the ability to perform each step successfully starting with accurate targeting of the desired anatomy. The implementation of the ClearPoint navigational software in this study allowed us to plan a safe trajectory through the parenchyma to reach our preselected target with a sub-millimetric accuracy of less than 0.2 mm radially, which is important when targeting small anatomical regions. In fact, intraparenchymal dosing of AAV2-GFP using two different infusion volumes was well tolerated with no adverse events noted during the survival period. In addition, we were able to actively control infusion parameters including rate, depth, and cannula position throughout the procedure. Equally importantly, the approach yielded a novel 3D imaging approach to showcase drug distribution and protein expression.

The fluorescent reporter distribution was consistent with the known AAV2 tropism for neurons and its ability to undergo axonal transport along established white matter tracts [25]. These findings reinforce the thalamus’s utility as a highly connected relay for achieving widespread cortical coverage, which is critical for gene therapy applications targeting neurodegenerative diseases [8,9]. Moreover, we leveraged CFT, a novel imaging approach, to map transgene expression across the whole brain. As previously shown in small animal models [14], CFT provides complementary data to conventional histological and analytical approaches. This is the first report to apply CFT at the whole-brain scale in NHPs, revealing robust thalamocortical transport of the vector not only to the overlying cortex, but also ventrally to the brainstem. Unfortunately, no spinal cord sections were collected at the end of the study to verify transport to the spinal cord.

As demonstrated in this study, MRI-guided neuro-navigation offers precise control over the infusion process, enabling surgeons to make real-time adjustments such as starting, stopping, modifying the infusion rate, advancing the cannula, and anticipating the extent of gene expression. Continuous MRI scan acquisition allows for immediate detection of brain shift, hemorrhage, or delivery complications, facilitating timely procedural corrections [20,26,27,28]. Techniques such as “infuse-as-you-go” further refined delivery in real time, contributing to a more streamlined and efficient procedure [24]. The flexibility of this method was highlighted by our ability to vary infusion volumes (85 vs. 170 μL) without compromising targeting accuracy or distribution. Notably, the total thalamic volume was effectively covered by a single infusion trajectory, with distribution extending across medial-lateral axes, a promising finding for translating this approach to clinical settings. Differences in GFP expression were evident between the two methods used in this study, CFT and histology, with the latter providing a more focal cellular specificity. Whereas, CFT provided a more global spread, consistent with other reports indicating greater cortical transport dependent on the total amount of the viral payload [29], axonal transport and directionality highlighting a dose-dependent effect [30].

The broader clinical relevance of this approach lies in its seamless applicability to both large animal models and future human trials, thereby enhancing translational continuity. The conserved connectivity of the thalamus makes it a promising target across species [31]. The integration of real-time imaging and whole-brain tools like CFT offers a reliable framework for reproducible gene delivery. While histology remains essential, CFT provides complementary data that can streamline analysis and support multiplexed visualization.

In summary, this small proof-of-concept study highlights a clinically relevant, MRI-guided gene delivery approach capable of achieving widespread thalamocortical expression using AAV2 vectors. The integration of real-time surgical control, advanced imaging modalities, and whole-brain analysis tools represents a powerful strategy to improve the precision, reproducibility, and translational value of CNS-targeted gene therapies. We recommend this approach for delivering AAV therapies targeting neurodegenerative diseases affecting deep brain regions.

## Figures and Tables

**Table 1:** Automated projected radial and depth error calculation.

Metric	Animal #1	Animal #2	
	Right Side	Right Side	Left Side
Projected Radial Error	0.7 mm	0.3 mm	0.5 mm
Actual Radial Error	0.6 mm	0.2 mm	0.3 mm
Difference in Radial Error	0.1 mm	0.1 mm	0.2 mm
Final Depth Error	−0.1 mm	0.6 mm	−0.2 mm

**Table 2:** Summary table of the most prevalent anatomical regions transduced by AAV2-GFP.

Brain Region	Structures Transduced	GFP Expression
Cerebral gray/white matter	Axons, neurons	Strong, 1 or 3 *
Internal capsule	Axons	Strong, 1 or 3 *
Lateral ventricle	Ependymal cells	Moderate, 1
Hypothalamus	Axons, neurons	Light to strong, 2
Thalamus	Axons, neurons	Light to strong, 2
Caudate nucleus	Axons	Light to moderate, 1
Pons	Axons, neurons	Light to strong, 2

Key: score of 1 = <5% cellular transduction; 2 = 5–10%; 3 = 11–50% and 4 = >50%. * Transduction score was higher based on the location of section examined.

## Data Availability

The original contributions presented in this study are included in the article. Further inquiries can be directed to the corresponding author.

## Abbreviations

The following abbreviations are used in this manuscript:

AAALAC	Association for assessment and accreditation of laboratory animal care
AAV	Adeno-associated viral
AAV2	Adeno-associated viral serotype 2
CED	Convection-enhanced delivery
CFT	Cryo-fluorescence tomography
CMV	Cytomegalovirus
CNS	Central nervous system
CSF	Cerebrospinal fluid
DAB	3, 3′-Diaminobenzidine
ECG	Electrocardiogram
EMA	European medicines agency
FDA	U.S. Food and drug administration
GFP	Green fluorescent protein
IACUC	Institutional animal care and use committee
MRI	Magnetic resonance imaging
ms	milliseconds
NHP	Nonhuman primate
OCT	Optimal cutting temperature
ROI	Region-of-interest
T1w	Time-1-weighted
